# A real-time field bus architecture for multi-smart-motor servo system

**DOI:** 10.1038/s41598-024-53022-2

**Published:** 2024-02-16

**Authors:** Zhichao Huang, Song Qiu, Bangji Wang, Qingxiang Liu

**Affiliations:** School of Physical Science and Technology, Southwest Jiaotong University, Chengdu, 610031 China

**Keywords:** Electrical and electronic engineering, Engineering

## Abstract

The multi-motor servo system (MMSS) is an electro-mechanical system widely used in various fields, including electric vehicles, robotics, and industrial machinery. Depending on the application, the number of motors in the system can range from several dozens to tens of thousands, which imposes additional communication demands. Thus, ensuring synchronization and control precision of the system requires addressing the challenge of guaranteeing the performance and reliability of communication among motors in the MMSS. In this paper, we design a smart servo motor (SSM) to upgrade the system to the multi-smart-motor servo system (MSMSS) based on a distributed real-time field bus architecture, namely, Multi-Motor Bus (MMB) architecture. The proposed MMB architecture is lightweight and stable, providing real-time support for Control Area Network connections to a central user computer and inter-integrated circuit connections to SSM units. This MMB architecture facilitates the synchronization of command transmission across SSMs and ensures the consistency of motors in the MSMSS. Additionally, a serial experiments to examine 3 key system performance and reliability characteristics are conducted, including command transmission time, transmission jitters, and rotation consistency. The analysis of these characteristics demonstrates the system’s potential and feasibility to be applicable in industry.

## Introduction

Servo motors are widely used in various industrial applications nowadays. With the increasing demands of modularization, miniaturization, and integration in applications, multiple servo motors can form a system to realise complex and ingenious functions. Such system with multiple servo motors is named as multi-motor servo system (MMSS)^1^. The MMSS is widely employed in various applications due to its attractive features, such as high efficiency, fast response of servo motors^2^. MMSS is not only used in automotive applications such as tunnel boring machine^3^, web-winding systems^4^ and electric vehicles^5^, but also plays a crucial role in heavy mechanical and highly integrated applications such as robotics^6^, cellular conveyor^7^ and electronically scanned arrays^8^. For the applications mentioned above, the performance and reliability demands usually lie with the rapid response capabilities, precise rotational abilities, synchronization control of the motors, and high robustness of the communication carrier within the MMSS. The communication carrier in the MMSS normally utilises wired means, that is, using conductor-wire to physically connect servo motors and control units to transfer the control commands and feedback information via communication bus protocol. Such communication strategy is usually the key to realise the high-precision and real-time control to the large number of motors inside the MMSS^9^. Therefore, it is important to focus on designing the communication strategy for the MMSS, examining the performance of the communication carrier chosen to be deployed in the MMSS, and guaranteeing the synchronization of system.

### Communication strategy used in the MMSS

With the widely use of the MMSS, the number of servo motors connected in a MMSS can range from dozens ($10^1\sim 10^2$) in electric cars^5^ and robotics^10^, to tens of thousands ($10^2\sim 10^4$) in cellular conveyor^7^ and radial line helical phased arrays^8^. With the increase of motors in the MMSS, the communication process between control systems and motor units, as well as between individual motors, can be highly intricate. To ensure proper operation of such a large number of motors, a suitable communication strategy should be carefully considered, taking into account the performance requirements of the MMSS. Several communication strategies with different bus protocols have been selected for MMSS, such as Recommended Standard (RS) 485 bus, RS 232 bus, Ethernet, and Control Area Network (CAN) bus. Each of them has its own features as shown in Table 1 and is suited to specific application domains. As a differential protocol, RS-485 is widely used in the remote control of industrial settings due to its long transmission distance and stability^11^. However, the number of nodes accommodated in the RS-485 protocol is limited into 32 nodes, which makes it difficult to meet the growing demand for an increasing number of motors in large MMSS^12^. As for the RS-232, it is widely available on computers and measurement equipmentS for short distance^13^. However, due to its low data transmission speed, short transfer distance, and point-to-point transmission characteristics, RS-232 can only be used as a branch of the communication component of a system for some non-time critical functions. As the next-generation network protocol increasingly adopted in vehicle gateways, Ethernet can provide bandwidth exceeding 100 Mbps, satisfying the real-time requirements of time-critical systems^14^. However, Ethernet tends to be a more expensive physical-layer interface and requires costly technology such as routers and switches, which increases the complexity and cost of network deployment in MMSS. Compared with the wired communication bus protocols mentioned above, the CAN bus not only incorporates some of their advantages, but also optimizes certain performance aspects^11–16^. As a multi-master protocol, CAN bus allows multiple devices to transmit data with each other, and also provides a reliable transmission mechanism due to its robust error detection mechanisms. Therefore, for complex working environments and an increasing number of electric motors, the CAN bus is better to meet the system’s communication requirements and achieve consistent control of motors.

**Table 1:** Characteristics of different types of networks.

Technology	Range	Maximum nodes	Maximum data rate	Complexity
RS485^11,12^	1200 m	32	10 mbps	Low
RS-232^13^	15.24 m	2	1 mbps	Medium
Ethernet^14^	40 km	255	10 Gbps	High
CAN^15,17^	500 m	110	1 mbps	ow

As a kind of serial communication protocol, CAN was developed in the mid-1980s by Robert Bosch, to solve the communication problem of the point to point method^17^. Over the years, the CAN bus has demonstrated excellent stability and efficiency, making it a widely used network in MMSS such as automobile manufacturing and vehicle networks^18^. However, the rapid development of MMSS and the increasing volume of data have led to a significant increase in system complexity.

### Related technology approach used in the MMSS

Nowadays, several works have been conducted on MMSS based on the CAN bus that integrate numerous motors from various segments to achieve control of the entire system. Some systems employ Micro Controller Units (MCU) as controllers, achieving functionality through code compilation. This approach is highly flexible and capable of meeting various algorithmic requirements^19^. However, these codes are executed sequentially, resulting in lower real-time performance. When controlling a single motor, a MCU can meet real-time requirements. But if you want to scale up the system with more motors, it requires multiple MCUs interconnected, making the communication network of the system considerably complex^20^. Furthermore, some systems design their controller on Field Programmable Gate Array (FPGA). The parallel processing capability of FPGA, coupled with its modular design approach, not only greatly reduces the control cycle but also enables synchronous control of the motors^21^. However, this design approach places high demands on FPGA’s logic resources. Additionally, because it is designed purely in hardware, it leads to limited computational power, especially in floating-point calculations, which will stress the burden for network analysis^22^. To achieve a fast and precise response in MMSS, this paper proposes a design solution for multi-smart-motor servo system (MSMSS) on both MCU and FPGA with smart servo motors (SSMs). By combining these two elements, it maximizes the parallel processing capabilities of FPGA and the computational power of MCU. As shown in the Table 2, the distributed control structure of the MSMSS not only efficiently conserves the resources of the main controller but also exhibits excellent scalability.

**Table 2:** Characteristics of different technological approach of MMSS.

Technology approach	Performance	Reliability	Scalability
MCU^19–21^	Moderate	Good	Moderate
FPGA^19,21,22^	Good	Moderate	Good
Hybrid FPGA	Excellent	Excellent	Excellent

### Contributions and organisations

In order to further understand the command transmission process of the MMSS, and tackle the limitation based on the review of related work, we propose the MSMSS with a real-time multi motor bus (MMB) architecture. The main contributions of this work are in the following respects: (1) A SSM has been designed as a control component within the system. The SSM integrates driving, controlling, communication, and feedback capabilities, enabling it to independently perform assigned tasks without relying on external control resources; (2) A MMB architecture is proposed to expand the number of motor units by taking the heterogeneous gateway to connect the user command center and SSM units; (3) A series of performance and reliability tests are conducted to show the feasibility of the proposed communication network for interlinked SSM units. Particularly, real-time characteristics are evaluated through analysis of the transmission time, transmission jitter, and consistency of SSMs. The rest of the paper are organized as follows: In "Method" section, diverse aspects of the experimental setup, such as the design of the system architecture, the implementation of the hardware with the selection of relevant electronic components, and the testing procedures are explained in detail. In "Result analysis" section, the experiment result are presented and analyzed. And finally in "Conclusions" section, the proposed work is concluded that key performance evaluations are presented.

**Figure 1:**
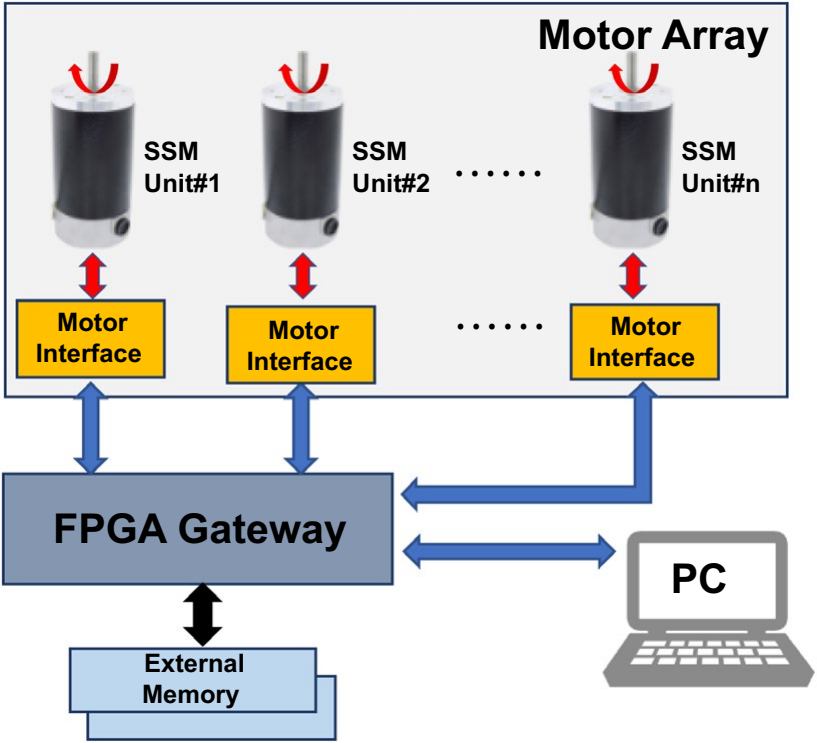
The architecture of the MSMSS based on the MMB architecture.

## Method

A typical MMSS comprises of a control panel that functions as the master of the system, and servo motors that serve as the system’s actuators^1^. The fundamental principles are also applicable to the our proposed MSMSS. However, in addition to these principles, conventional servo motors are replaced by SSMs, which possess the ability to drive, control, communicate, and feedback^23^. Furthermore, the direct connection between the master controller and motors is removed. Instead, a gateway is used as a medium to connect the user command center and SSM units, enabling further expansion of the system’s motor count. Figure 1 shows the layout of our proposed MSMSS with the SSM based on the MMB architecture.

### System architecture

**Figure 2:**
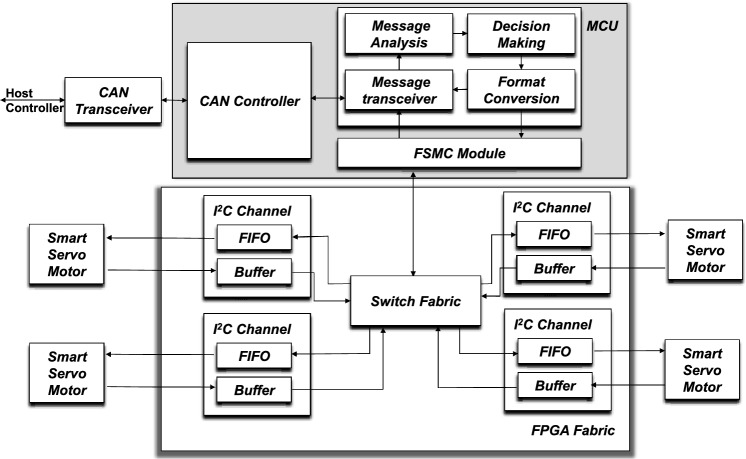
The architecture of the hybrid gateway.

To meet requirements of complex data analysis, a personal computer (PC) is used to as the command center to connected to the CAN bus via a USB-CAN adapter. With powerful calculation capability, the user command center can provide efficient delivery of key information or instructions to SSMs, such as the direction of rotation, the sequence of controlled motors, and the zero position of the motor, as well as managing data received from SSMs for analysis and investigation. To accommodate an increasing number of motors in the system, a hybrid gateway is added to the system as an internal transfer station that connects the user command center and SSMs. The gateway is designed using a hybrid of FPGA, which is composed of a MCU as an computation center and a FPGA fabric. Figure 2 shows the internal function connection of heterogeneous gateway. The MCU serves as the processing core of the gateway, which is equipped with CAN controllers and other hardware devices. Due to the high clock frequency and abundant peripheral resources, MCU has sufficient computing capability to execute complex algorithms, including receiving, parsing, classifying, and converting data. The instructions resulting from this processing are transmitted to the FPGA frabric via the Flexible Static Memory Controller (FSMC) module. As a parallel bus, FSMC significantly reduces the communication latency between the MCU and FPGA. After receiving the instructions, the switch state machine within the FPGA distributes these instructions to various SSMs connected to different ports via the inter-integrated circuit (I2C) bus. By exploiting the parallel processing capabilities of the FPGA and the processing capability of MCU, the gateway distributes the received instructions from the computer to the SSMs, while simultaneously ensuring the synchronization of data transmission and precision control of SSMs.

**Figure 3:**
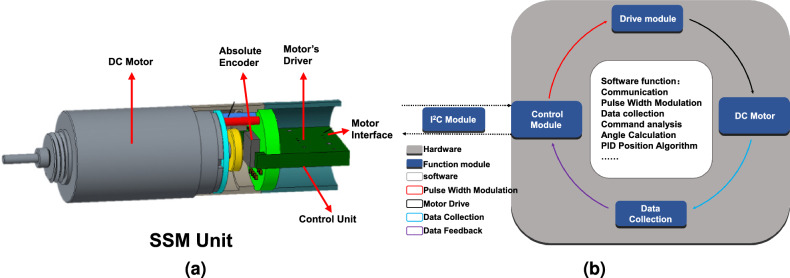
(**a**) The structure diagram of SSM unit, (**b**) The diagram of SSM functions.

**Figure 4:**
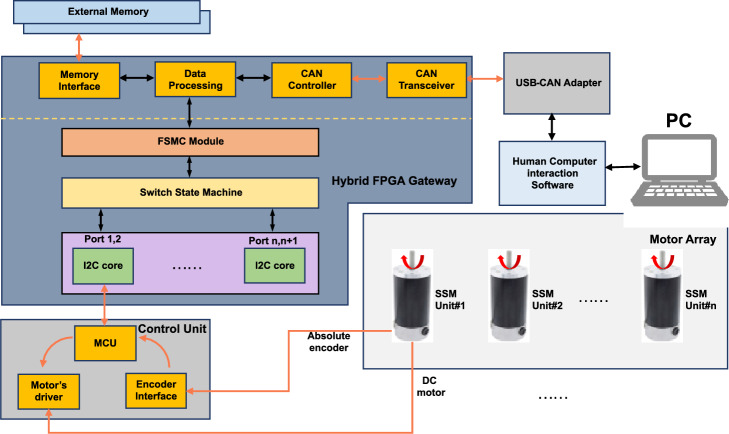
Layout of the MSMSS.

As the control object of the MSMSS, SSM is a relatively independent entity equipped with a control unit, which is highly integrated with a micro-controller, a driver chip, and a magnetic encoder as shown in Fig. 3a. As the core component of SSM, we have employed a 32-bit MCU as the primary controller at the bottom of the motor. The controller operates at a frequency of 180MHz and possesses abundant peripheral resources. This configuration not only enables precise motor control and rapid response but also leaves room for future upgrades. As shown in the Fig. 3b, the overall structure of the MCU functions forms a closed-loop system, in which various modules progress step by step to collectively accomplish the process from data reception and motor control to the return of working information. Upon the receiving message from the gateway via the I2C bus, the MCU determines the type of command and starts up the position-speed PID algorithm to produce pulse width modulation (PWM). To achieve rapid response and precise control of the system, the modulation frequency for both is 10KHz and 200KHz, respectively. Through a timer, the main controller continuously invokes the PID algorithm to adjust the driver’s output and control the motor’s rotation. To ensure the accuracy of rotation and collect SSM’s status, we have designed an absolute magnetic encoder, which is placed at the rear of the motor. The rotation of the motor drives the rotation of the magnetic ring at the rear, resulting in changes in the magnetic field. The encoder obtains information about the motor’s rotation based on changes in the magnetic field and provides feedback to the MCU to adjust PWM output, enabling closed-loop control. After all of functions have been added to hybrid FPGA gateway and SSMs, the layout of the our proposed SSM system is shown in the Fig. 4.

### System prototype

Based on the MSMSS proposed in the Fig. 4, we have built a prototype to demonstrate the function and performance of the system. The prototype consists 3 key components: (1) a user command center of the system, (2) a hybrid FPGA gateway, (3) a number of SSMs. Through the USB-CAN converter, the computer operates as a node of the CAN bus, with the ability to transmit and receive CAN frames through the user interface. As for the gateway, We have designed a hybrid circuit board used for gateway development which consists of two main components: a MCU(STM32F429) and a Altera Cylone 4 FPGA chip. The MCU is used as a computation core of the gateway, which is responsible for the analysis of the commands. And the FPGA chip has 22320 logic cells, which is equipped with abundant hardware resource for gateway function development.

The SSM is composed with a motor and a control unit. The motor used is DC micro motor, which is cost-efficiency and has rapid response^24^. The control unit consists 3 component, MCU, driver chip and magnetic encoder.The controller uses STM32F042 based on the Cortex-M0, which is convenient to be deployed and provide sufficient resource to execute complex algorithm. The motor driver chip is from DRV series, which provide integrated motor driver performance for low-voltage motion control applications. As for magnetic encoder, an absolute encoder, MT series, is taken to collect the information of motor, which provide 14 bits resolution of angle. Table 3 summarizes the parameters of the components used in our experimental prototype.

**Table 3:** The experimental prototype and components’ parameters.

Component	emphFeature	Parameters
Hybrid gateway	Micro-controller chip	STM32f049
	CPU core	Cortex-M4
	Dominant frequency	180 Mhz
	FPGA	EP4CE22F17C8N
	Logical resource	22320
	Max user I/O	153
SSM unit	Micro-controller chip	STM32F042
	CPU core	Cortex-M0
	Dominant frequency	48 Mhz
	Motor driver	DRV series
	Max current	1.8 A
	Absolute encoder	MT series
	Angle resolution	14 bit

**Figure 5:**
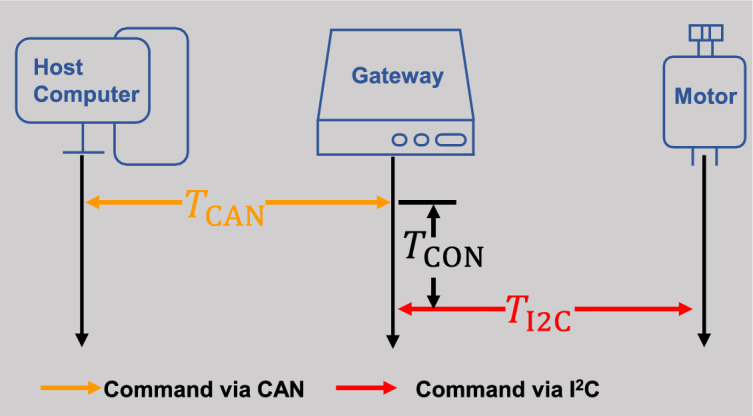
Packet route of the test topology.

### Evaluation process

To analyze the performance parameters of MSMSS, we conduct series evaluations to create sufficient results for out analysis under a predefined command pool. The aim of evaluations is to measure transmission time(TT) and jitter of packet for latency demonstration and record the motion movement of the SSMs to evaluate the control precision and consistency of the system. During the evaluation, we have programmed a user command center to control movement of SSMs in the CAN standard format and selects 6 commands to verify the performance of the system by time-trigger approach. Figure 5 illustrates the packet routes during the communication in the experiments. As for the packets used in the experiments, they are selected messages from parameter groups(PGs) with different priority as shown in the Table 4. Each piece of message corresponds to a control instruction, such as communication selfcheck, multi motor rotation, etc.

**Table 4:** CAN message of the command pool.

Priority	PGN	Transmission rate	Data length
1	0	10 ms	64 bits
5	P01	100 ms	64 bits
6	P60	40 ms	64 bits
6	P61	100 ms	64 bits
8	P08	50 ms	64 bits
9	P09	250 ms	64 bits

#### Communication performance

**Figure 6:**
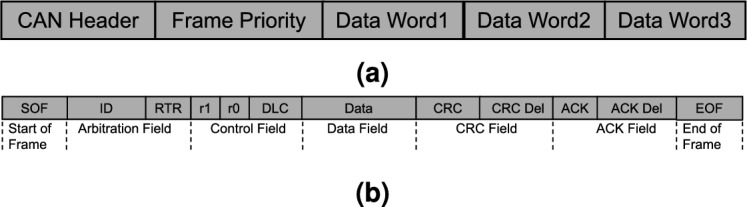
(**a**) Command Structure used in the MSMSS, (**b**) Standard CAN Frame Format.

The transmission time of commands is a crucial metric that indicates the latency of a communication process, which refers to the time delay for the successful end-to-end transmission of a single test packet. This metric is composed of several factors, including the worst-case response time of the CAN frame $T_\text {CAN}$, the conversion time of the gateway $t_\text {CON}$, and the transmission time of the I2C frame $T_\text {I2C}$ as follows:

1 $$\begin{aligned} T_\text {TT} = T_\text {CAN} + T_\text {CON} + T_\text {I2C}, \end{aligned}$$

where $t_\text {CAN}$ represents the time required for a CAN frame command to be generated, queued, transmitted, and ultimately received by the gateways, $T_\text {CON}$ represents the time required for analysing commands and converting formats in the gateway, $T_\text {I2C}$ is the time for the packet transmission via the I2C bus from the port of gateway to the terminal of SSMs. To gain a deeper understanding of the transmission process of data within the system, we conduct theoretical analysis of the three phases. Firstly, as the bridge for communication between the user interface and the MSMSS, the worst case response time of CAN frame $T_\text {CAN}$ can be calculated by the Tindell equation^25^ as follows:

2 $$\begin{aligned} T_\text {CAN} = W_\text {m} + C_\text {m}, \end{aligned}$$

where $W_\text {m}$ represents the longest time interval between placing message in queue and the start of transmission. $C_\text {m}$ is the time taken to transmit message on CAN bus. Figure 6a shows the structure of command message used in the system, and Fig. 6b is the format of the CAN message. According to the format of a standard CAN frame, $C_\text {m}$ can be defined as follows:

3 $$\begin{aligned} C_\text {m} =\left( \frac{[34 + 8S_\text {CAN}]}{5} + 47 + 8S_\text {CAN}\right) {\tau _\text {CAN}}, \end{aligned}$$

where $S_\text {CAN}$ represents the number of the data bytes within the CAN message, $\tau _\text {CAN}$ is the time taken to transfer one bit on the bus.

**Figure 7:**
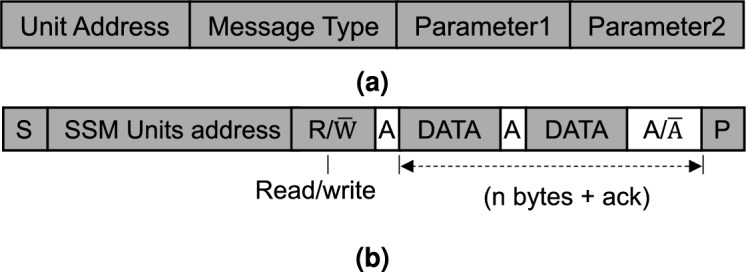
(**a**) Converted I2C structure, (**b**) I2C frame format.

With respect to the duration $T_\text {CON}$ required for the transmission of the command within the gateway subsequent to its arrival, it includes not only the time taken for the command analysis but also the conversion time between the CAN frame and I2C structure. The primary determinant of the duration $T_\text {CON}$ is the frequency of the system clock as well as the level of optimization of the algorithm utilized. According to the I2C structure mentioned in the Fig. 7, the converted I2C frame information primarily consists of a 7-bit address segment, 8-bytes data segments, and the response signals between each byte, so the $T_\text {I2C}$ can be defined as follows:

4 $$\begin{aligned} T_\text {I2C} =(7 +Ack_\text {I2C}+8S_\text {I2C}){\tau _\text {I2C}}, \end{aligned}$$

where $Ack_\text {I2C}$ represents the number of responses between the master and slave, $S_\text {I2C}$ represents the quantity of byte data segments, and $\tau _\text {bit}$ represents the time required for transmission of a bit in I2C communication.

After analysing the command transmission process in the system, we can assess the real-time characteristics of the system by using the transmission time. For a fixed-priority system, we can employ the transmission factor to characterize the real-time characteristics of the system. Assuming that there are a set of instructions in the system, we use $T_\text {1}$, $T_\text {2}$, ..., $T_\text {n}$ to donate the transmission period of n pieces of command messages with their transmission time $T_\text {TT,1}$, $T_\text {TT,2}$, ..., $T_\text {TT,n}$, respectively. Thus, the transmission factor can be calculated as follows:

5 $$\begin{aligned} U = \sum _{i=1}^{n} \frac{C_i}{T_\text {TT,i}}, \end{aligned}$$

where *U* represent the transmission status of a set of periodic messages in the system. We can obtain the boundary condition for transmission time iteratively, as represented by the following equation^26^:

6 $$\begin{aligned} U \le n(2^{1/n}-1), \end{aligned}$$

where *n* represent the number of messages in the system. A smaller value of transmission factor *U* indicates better real-time performance of the system.

#### System consistency

In order to demonstrate the consistency of our MSMSS prototype and the synchronization of SSMs, we focus on evaluating the following metrics: the responding time of commands (RTC), the rotation precision of angles (RPC), and the consistency of motion movement (CMM). RTC represents the duration between the motors starting to operate and achieving the functional target, which reflects the responding speed of SSM units. RPC represents the degree of task completion, indicating the precision of SSM units. Finally, CMM represents the consistency of SSMs under the MSMSS, which is a vital metric for reflecting the cooperation condition among motors.

## Result analysis

**Figure 8:**
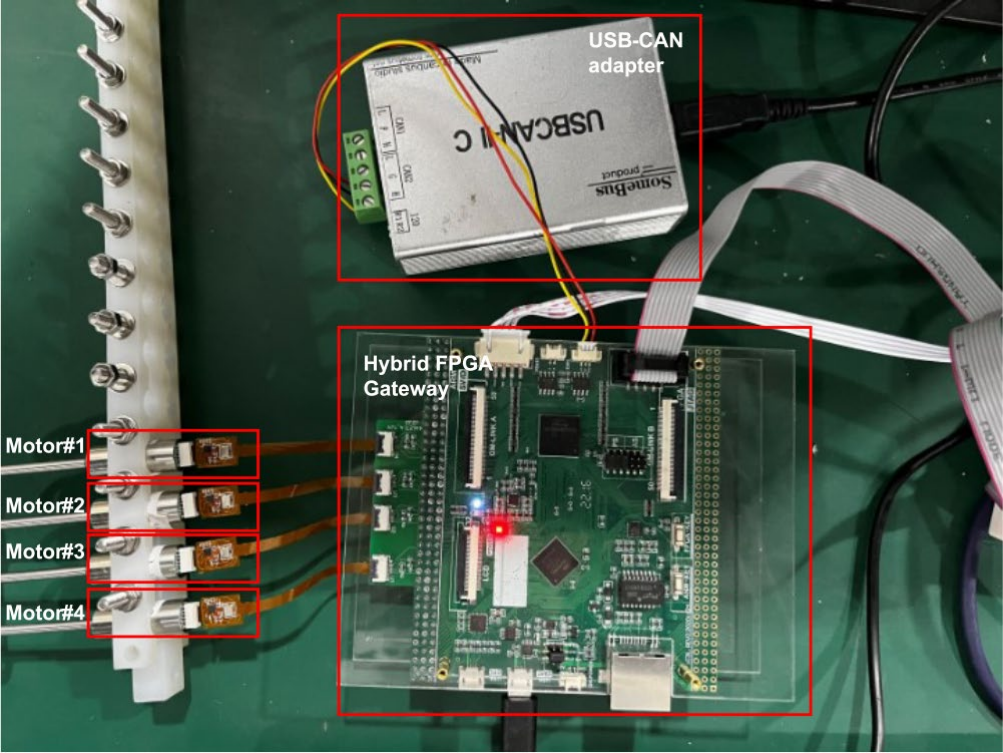
The prototype in the MSMSS.

To verify the performance and the synchronization of the MSMSS, we have built a prototype as shown in the Fig. 8. The MSMSS is composed with 4 SSMs and a hybrid gateway, which is controlled by a computer. We have conducted a series experiment to create sufficient results for analysis. The real-time characteristics of system are discussed based on the evaluation results. Afterwards, the metrics of performance and the consistency are analysed for the reliability and performance of our proposed MSMSS.

### Transmission time

**Figure 9:**
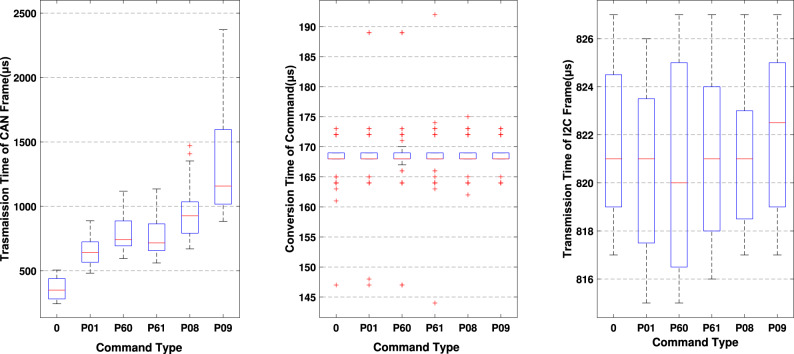
Transmission time: (**a**) Transmission time of CAN frame, (**b**) Conversion time, (**c**) Transmission time of I2C frame.

As a crucial metric that indicates the latency of a communication process, the whole transmission time is composed with 3 parts of time, CAN transmission time $T_\text {CAN}$, conversion time of CAN frame and I2C frame $T_\text {CON}$ and I2C transmission time $T_\text {I2C}$. The Fig. 9 separately analyses the transmission time of commands with 6 different priority through a time-trigger approach. With the decrease of the priority, the transmission time increases. According to the Eq. 3, the $T_\text {CAN}$ consists of two parts $W_\text {m}$ and $C_\text {m}$. Since the data segment length of the instruction is 64 bits, the $C_\text {m}$ of commands is essentially constant based on the Eq. 2. Thus, the fluctuation of $T_\text {CAN}$ primarily arises from the queuing time of commands between different priority levels. As the highest priority command message, the mean transmission time $T_\text {CAN}$ of PGN 0 is approximately 300$\upmu {\hbox {s}}$, making it the shortest transmission time among the message pool. This is approximately 1.5 times shorter than the mean transmission time of PGN P01, 2 times shorter than the mean transmission time of PGN P60 and PGN P61, 3 times shorter than the mean transmission time of PGN P08, and 4 times shorter than the mean transmission time of PGN P09. Compared the transmission time of different CAN message in the MSMSS, it is important to note that the range of transmission time of message is also increasing which means the increasing of the jitter for different priority CAN message during the transmission.

AS one part of TT in the MSMSS, the conversion time from CAN frame to the I2C frame also reflects some transmission characteristics. According to the Fig. 9b, the conversion time $T_\text {CON}$is stable at around 160$\upmu {\hbox {s}}$ no matter what kind of priority CAN message are.This is because during the conversion process, the instruction parsing takes place in the MCU. The parsed data is synchronized and processed in the FPGA in parallel, converting it into I2C frame.The priority of commands will not exert an influence on the entirety of the process. The time required for the conversion process is determined by the length of the information and is independent of the type of information. After the conversion progress, the control commands assigned to the corresponding port and then transmitted into SSMs in I2C format. As shown in the Fig. 9c, the $T_\text {I2C}$ is stable around at 815$\upmu {\hbox {s}}$. Because the parallel characteristics of the gateway, these I2C frames do not need to queue or compete with each other. Thus, no matter the I2C message converted from what kind of the CAN frame, the transmission time of I2C messages is approximately the same.

Thus, based on the Eq. 1, we can summary the entire transmission time of command in the MSMSS. As shown in the Table 5, the minimum $T_\text {TT}$ in the command pool is PGN 0, which is about 1108$\upmu {\hbox {s}}$ from the time command is generated to the time command arrived at MSMSS. And the maximum $T_\text {TT}$ is CAN frame with the lowest priority, the PGN P09, which takes 3369$\upmu {\hbox {s}}$ when the network is under busy condition. By substituting the maximum transmission time and trigger periods of each command into Eq.5, we can obtain a value of transmission factor *U* as 0.31, while the system’s boundary condition in Eq. 6 is 0.73. By comparing the values of *U* with the boundary condition, we can observe that the *U* is significantly smaller than the boundary condition, with a numerical value that is only half of the boundary condition. This implies that the completion of system instruction transmission is excellent, and the system exhibits good real-time characteristics.

**Table 5:** Transmission time of command in the MSMSS.

Message PGN	Total transmission time		Message jitter
	Min	Max	
0	1108 us	1471 us	3463 us
P01	1444 us	1811 us	367 us
P60	1523 us	2135 us	612 us
P61	1555 us	2137 us	582 us
P08	1649 us	2473 us	824 us
P09	1864 us	3369 us	1505 us

### Jitter of communication

Jitter is an important parameter for real-time systems, which reflects the variability in the system response time. Table 5 shows the maximum and minimum transfer time for the message in the command pool from the moment the message queued for transference to the moment they arrive at the SSMs in the network. By subtracting the maximum and minimum values of transmission time, we can obtain the jitter of each command during the transmission process. As seen in the Table 5, the PGN 0 has the shortest jitter, primarily due to its highest priority in the communication network. Based on our previous analysis of the communication network, we can conclude that the most significant factor affecting the transmission time in the system is the queuing time $W_\text {m}$ for command. when multiple commands are waiting in the queue of the bus for transmission, they compete with each other, resulting in the generation of jitter. That is why the highest priority command PGN 0 has the shortest jitter, which makes it always transmitted in the first place, while PGN P09 has the longest jitter.

**Figure 10:**
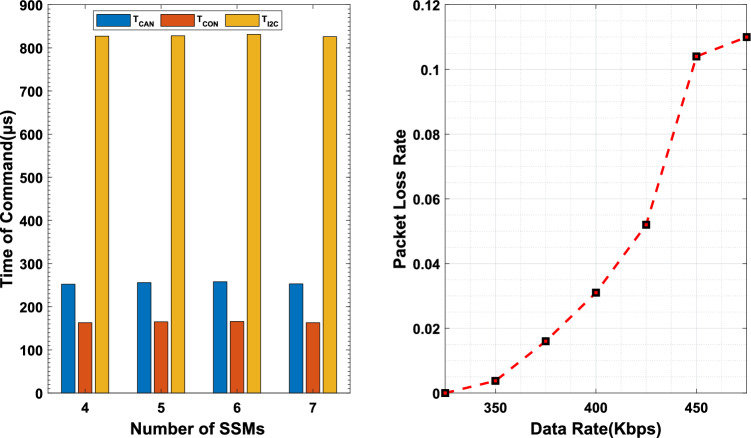
Performance of the communication network: (**a**) Scalability of the network, (**b**) Reliability of the network with 500Kbps bandwidth.

**Figure 11:**
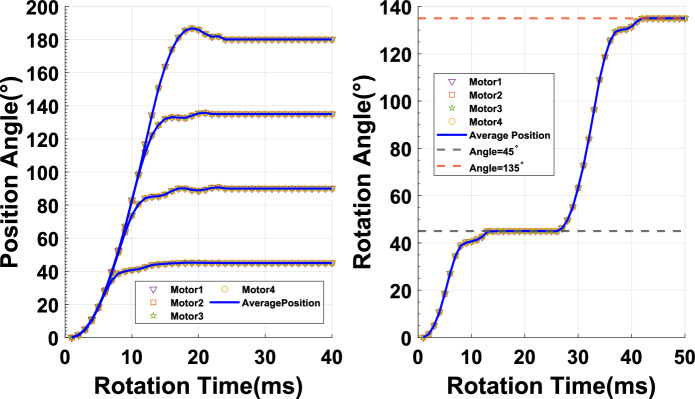
Rotations performance of SSM: (**a**) Multi-angle position control, (**b**) Sudden position change control.

### Reliability of network

To meet the increasing trend of the number of motors and the need for synchronization control among motors, the reliability of the communication network within the system is of paramount importance. While sending commands to the system, we sequentially increased the number of motors in the MSMSS. At this point, there was only rotation command PGN P61 in the transmission network, so there was no competition on the bus. As shown in the Fig. 10a, as the number of SSMs within the system gradually increased, the transmission time of instruction in various parts of the system, $T_\text {CAN}$, $T_\text {CON}$, $T_\text {I2C}$, remained stable. This is because MSMSS employs parallel control, utilizing the parallel capabilities of the FPGA to transmit instructions to the SSMs. Additionally, SSMs operate independently of each other, without mutual interference. Thus, an increase in the number of SSMs does not impact the performance of the communication network. Figure 10b shows the packet loss rate of the network. As the input data rate of the system increases, packet loss rate gradually rises. Especially when the input data rate approaches 500Kbps, the packet loss rate experiences a steep increase. This is because, in addition to the input data, MSMSS also generates feedback information. The combined data volume of both has exceeded the system’s bandwidth capacity of 500Kbps, causing communication congestion and an increase in the data loss rate. However, for a system with infrequent triggers, where the data rate always remains below 350Kbps, the packet loss rate of MSMSS remains consistently below 0.2%. The stable transmission time and a 0.2% packet loss rate indicate excellent scalability and reliability of the system.

**Table 6:** CAN message of the command pool.

	Hybrid FPGA	FPGA^27^	MCU^28^
Position	45$^{\circ }$	45$^{\circ }$	45$^{\circ }$
Response time	20 ms	50 ms	60 ms
Overshoot	0.8%	4.1%	6.3%
Steady-state error	0.25$^{\circ }$	1.4$^{\circ }$	1$^{\circ }$
Logical resource	0.2 K	2.5 K	None

### Performance and reliability

After receiving the command from the gateway, SSMs start to execute the function and incorporate with each other. We focus on SSMs’ performance and reliability, such as RTC, NPC, and CMM, and evaluate the performance of the proposed MSMSS. Therefore, we have recorded the rotation condition of SSMs under rotation command including individual motors rotating at multiple angles and multiple motors rotating at the same angle.

#### Response time and rotation performance

As shown in the Fig. 11a, SSMs were demanded to rotate for 45$^{\circ }$,  90$^{\circ }$,  135$^{\circ }$ and 180$^{\circ }$, respectively. The response results of different target positions indicate that SSMs can achieve synchronous responses within 180 degrees. The RTC of SSMs is less than 20ms from the motors’ response to the rotation command the completion of the rotation. Additionally, the SSMs’ position trajectory remains relatively smooth. The RPC of SSMs is approximately 0.25 $^{\circ }$, which reflects the difference between the stabilized angle of the motor and the target angle fluctuates. Figure 11b analyzed the position trajectory of the SSMs during a position step change. During a position step change, the SSMs’ rotational process remains smooth without significant overshoots. According to the results of the Fig. 11, our proposed SSMs not only exhibits fast response time, but also high rotational precision. This can be attributed primarily to our compact hardware structure, faster modulation cycles, and precise encoder feedback. In Table 6, we presented the performance of SSM in terms of response time, overshoot, steady-state error, and logical resource. Compared with motor controlled by the MCU^28^ or FPGA^27^, SSM exhibits faster response times, lower overshoot, and smaller steady-state errors, which can achieve control more smoothly and quickly.

**Figure 12:**
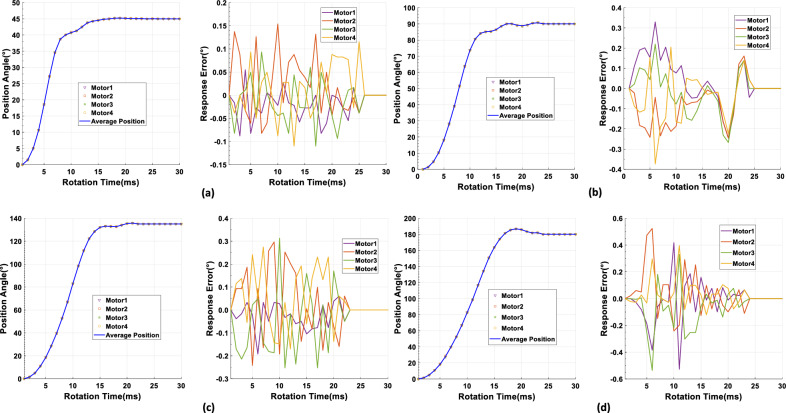
Synchronization control of MSMSS: (**a**) Consistency of multi SSMs for 45$^{\circ }$ and response error, (**b**) Consistency of multi SSMs for 90$^{\circ }$ and response error, (**c**) Consistency of multi SSMs for 135$^{\circ }$ and response error, (**d**) Consistency of Multi SSMs for 180$^{\circ }$ and response error.

After qualifying the performance of the SSMs, we conducted experiments to rotate 4 SSMs to verify the consistency of the system for complexity and synchronization requirements. we sent the rotation command PGN P61 to 4 SSMs for rotating 45$^{\circ }$, 90$^{\circ }$, 135$^{\circ }$ and 180$^{\circ }$. We compared the rotation trajectories of the four SSMs with the average position curve and analyzed the response error of the system during the rotation process in the Fig. 12. All SSMs responded simultaneously and completed the rotation in approximately 20ms. The position trajectories of system highly overlapped, which indicated a high degree of consistency among the motors. As the rotation angle increases, the response error gradually increases. As shown in Fig. 12a, when rotating 45 $^{\circ }$, the system’s position trajectory is very smooth, and the response error remains within 0.15 $^{\circ }$. When the rotation angle reaches 90$^{\circ }$, the entire trajectory curve remains smooth, but the response error increases to 0.3 $^{\circ }$ in the Fig. 12b. Figure 12c records the system’s response when it rotates 135$^{\circ }$. There isn’t significant fluctuation during the rotation process, and the response error remains within 0.3 $^{\circ }$. Until the system reaches 180 $^{\circ }$ of rotation, the system’s position curve exhibits a considerable overshoot, exceeding the target angle by 2 $^{\circ }$ in the Fig. 12d. This is due to the PID algorithm causing an excessive output magnitude during the adjustment process. The maximum difference in the rotation trajectory among the SSMs remains at around 0.5$^{\circ }$  indicating that the SSMs of the system exhibits excellent consistency and synchronization.

#### Stability under different temperature environment

**Figure 13:**
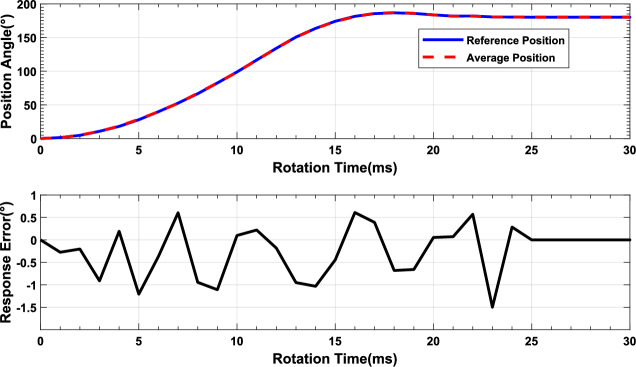
The low temperature reliability of MSMS: (**a**) Consistency of MSMS under -20$^{\circ }{\hbox {C}}$, (**b**) Response errors.

**Figure 14:**
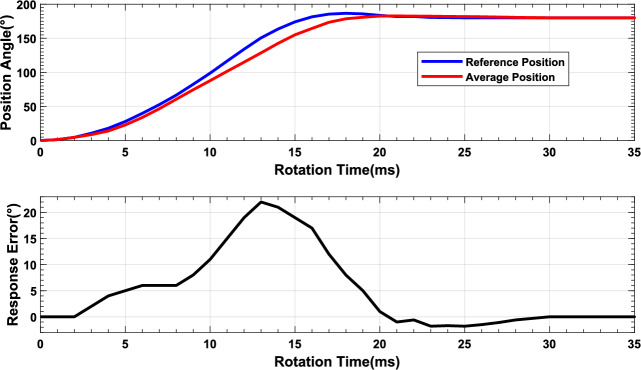
The results of the high and low temperature reliability tests: (**a**) Rotation condition under $-$ 20$^{\circ }$C, (**b**) Response errors.

To further validate the applicability of the MSMSS in industrial application, robustness tests were conducted to evaluate the system’s performance in high-temperature and low-temperature working environments with China National Standards^29^. In this tests, the MSMSS was deployed in both low-temperature (i.e., $-$20$^{\circ }$C) and high-temperature (i.e., 85$^{\circ }$C) environment, which was close to the extreme working temperature conditions of the DC micro motors. We send 180$^{\circ }$  rotation command to the MSMSS to observe 4 SSM units operation results, and compared the average position curve with the reference position curve which is the average position under normal temperature. As shown in the Fig. 13a, the average position curve shown in red closely aligns with the reference position shown in blue. Figure 13b analyzes the response error between the average position curve and the reference position curve. The fluctuation in the difference of position is within 2 degrees, indicating that the entire system is highly reliable. Figure 14 illustrates the system’s control performance under high-temperature conditions. As the temperature increases, the performance of the SSMs begins to degrade. As shown in the Fig. 14a, b, compared to the trajectory curve of the system at room temperature, the MSMSS’s position trajectory is slower. Not only does the system’s position trajectory show a slower rising trend, but the system’s response time also increases. However, due to the system’s short modulation cycle and accurate feedback information, the system is still able to reach the target angle in 30ms and remain stable. It can be seen that although the MSMSS’s performance may decrease under high-temperature conditions, it can still complete the the control tasks.

## Conclusions

In this paper, we firstly reviewed the related works on multi-motor system that used for the industry domain, and addressed the importance to analyze the performance and reliability of communication network for the MSMSS. To quantify the performance and verify the feasibility of our proposed MSMSS and MMB architecture, we designed smart servo-motors that were interlinked with a hybrid gateway based on the FPGA, to guarantee the control command flow simultaneously within the system. In order to achieve such objectives, we conducted a series of evaluation based on MSMSS prototype in the laboratory. The experimental results demonstrated its capability to ensuring synchronized transmission within 5ms of comprehensive command messages, owing rotation precision within 0.25$^{\circ }$, maintaining rotation consistency among the SSMs motor units within 0.5$^{\circ }$, and exhibiting industrial applicability within motor working temperature conditions.

## Data Availability

All data generated or analysed during this study are included in this published article.

## Abbreviations

*T*_TT_: Transmission time of a command
*T*_CAN_: Worst -case response time of CAN frame
*T*_CON_: Conversion time of the gateway
*W*_m_: The longest queueing time of CAN frame
*C*_m_: The transmit time of CAN frame
*T*_12C_: Transmission time of l2C frame
*S*_CAN_: Number of the date bytes within the CAN message
*τ*_CAN_: Time required to transmit one bit on CAN bus
*Ack*_12C_: Response times between master and slave
*S*_12C_: Number of data bytes within the l2C message
*τ*_12C_: Time required to transmit one bit on l2C bus
*U*: Transmission factor
MMSS: Multi-motor servo system
MSMSS: Multi-smart-motor servo system
MMB: Multi-motor bus
FPGA: Field programmable gate arrays
CAN: Control area network
l2C: Inte-integrated circuit
SSM: Smart servo motor
PC: Personal computer
UI: User interface
MCU: Micro controller units
FSMC: Flexible Static memory controller
PWM: Pulse width modulation
PGs: Parameter groups
PGN: Parameter group number
RTC: Responding time of commands
RPC: Rotation precision of angles
CMM: Consistency of motion movement
